# Evil Effects of Mercury

**Published:** 1898-01

**Authors:** R. W. Starr

**Affiliations:** Chicago, Ill.


ARTICLE III.

### EVIL EFFECTS OF MERCURY.

BY R. W. STARR, D. D. S., CHICAGO, ILL.

For the past four years I have been endeavoring to persuade my friends in the dental profession to break away from narrow prejudices, and to investigate for themselves by due process of observation and experiment whether mercurial preparations in the mouth were not in many instances very injurious to the individual having them. The results of my efforts in that direction are not as satisfactory as I could wish; though in some cases enough interest has been awakened to, in a measure, compensate me for my labor.

Dentists are but human beings, and as such are liable to make many grievous errors, and frequently content to follow out a certain line of thought and action, simply because they were taught at college that such things were true and right.

How many of the theories and ideas of fifty years ago are looked upon by the enlightened people of to-day, as being so utterly ridiculous, that it seems impossible that any one should have ever tolerated them.

Are we of to-day any the less liable to err than our forefathers? I think not; therefore whenever a question arises it is much better to investigate the subject thoroughly and find out what is true for ourselves, than to decide and wrap ourselves in a mantle of conceit and self congratulations.

That a great many persons are to-day suffering from the effects of mercurial preparations in the mouth, in the forms of amalgams and red rubber plates, I am firmly convinced, six years of constant study and research in this direction having demonstrated to my satisfaction that such is the case.

There is no truer statement than that we are not at all times equally susceptible to the same things, a substance which may to day prove nourishing, may at some future time prove not only distasteful but absolutely injurious and even poisonous; all of which is due not to a change in the character of the substance, but to some developed idiosyncrasy of the individual.

The same is true of mercury. We may have it in the mouth for years without experiencing any bad effect, and later may develop a mercurial diathesis of which we are unaware, the result being we are under the influence of this most lasting drug, while we are in total ignorance of the fact.

I can hear the skeptical say, why this is impossible, there is not enough mercury in an amalgam filling to do any harm, and as for red rubber plates, never by any means has free mercury been obtained from it

In answer I would say, it is not the quantity but the ever presence that does the damage. With every mouthful of food, either liquid or solid, carried into the stomach minute particles of mercury goes down with it.

One peculiarity of its action is that any one suffering from a chronic ailment is much more susceptible to it than when they were not under chronic influence.

The effect upon the nervous system is marked and nearly always predominates.

I maintain that over one-half of those afflicted with what is commonly called facial neuralgia, can be greatly relieved by simply removing the mercurial preparation, and generally cured by antidoting the drug effect.

In throat troubles it is especially active. One case in mind : young lady æt. twenty, elocutionist, was repeatedly attacked by tonsilitis. When these came on, which were generally quite sudden, would lose her voice.

This condition lasting several days, making it impossible for her to keep her business engagements; had treated for it for three years, but beyond palliation could not make any advance.

She was finally sent to me by her physician, who had begun to be suspicious of her amalgam fillings. I removed twenty of these and replaced with oxyphosphate, later with gold. From the time the amalgam was removed, February 1, till the cavities had been refilled with gold, May 1, she had one attack; since then, two and one-half years, she has had no recurrence of the trouble. After I had removed her fillings her physician put her on medicine to antidote the mercury.

Another case: Mrs. B., æt. twenty-seven, had a vascular tumor on the gum, between the left central and lateral incisor ahout the size and shape of a large chestnut, which had been growing for eighteen months; she had it cut away and cauterized several times, but it always returned with renewed energy.

Her dentist had in despair recommended the extraction of the incisor teeth and cutting away of the alveolar process, as the only possible cure; this would necessitate the taking of an anæsthetic, which she would not consent to do, as she was then pregnant.

The tumor bled constantly and the hæmorrhage was worse at night than in the day, amounting to about two ounces of blood in twenty-four hours.

Her physician who was an old friend of mine, sent her to me to ask my opinion as to the necessity of an operation after a thorough examination and history of the case, recommended that the operation be postponed until last resort; removed eight amalgam fillings and antidoted the mercury, then a few days later cut away the tumor and cauterized thoroughly; at the expiration of two months all traces of the tumor had disappeared.

These are but two of many similar cases which I have had and cured of a reputed incurable disease; not that my medicine or treatment was any better or more thorough than others, but because I had eliminated a condition which was antagonizing all other drug effects, as mercury once it obtains foothold in the system will generally neutralize all other weaker drugs which may be administered. Any one who has had a patient who has been salivated, or taken much mercury in its many forms, will I think bear me out in this statement.

As to red rubber plates; if it is not the coloring matter but the nonconductivity of the rubber as some claim, which is injurious, why is it that such mouths will frequently give no trouble if black rubber is substituted; this has been done successfully many times.

I could cite many other cases but space will not permit.

For the benefit of those who wish to investigate this subject, I will state a few of the leading symptoms of mercurial effects; facial neuralgia, feeling of great lassitude, aching of the limbs, choking as if a lump in the throat, extreme sensitiveness to cold, desire to be warmer even when in perspiration, oppression as if a weight upon the chest, palpitation of heart upon a slight exertion, and profuse flow of saliva. Of these symptoms it is quite common to find five or six present in one case; though of course one can hardly expect to find them all at one time, still I have had several cases which did have all. Now I would like to ask what logical deductions are to be drawn from such experience?

If one has had such good results why cannot others? If I have succeeded in awakening in some of my readers a desire to investigate this subject thoroughly, I shall feel that I have been amply repaid.

Perhaps you will ask what I would suggest as a material for filling such cavities as are generally filled with amalgam; I would recommend gutta-percha, cement or tin where gold cannot be used, but at any rate use anything in preference to a material which contains mercury.

To those who are skeptical, who see more profit in derision than investigation, I would say that "none are so blind as those who will not see."—*Dental Review.*

